# Transcriptome-scale spatial gene expression in rat arcuate nucleus during puberty

**DOI:** 10.1186/s13578-022-00745-2

**Published:** 2022-01-21

**Authors:** Shasha Zhou, Shaolian Zang, Yanping Hu, Yifen Shen, Hua Li, Wenlian Chen, Pin Li, Yihang Shen

**Affiliations:** 1 Central Laboratory, Suzhou Ninth People’s Hospital, 2666, Ludang Road, Suzhou, 215200 Jiangsu China; 2 Department of Endocrinology, Shanghai Children’s Hospital, Shanghai Jiao Tong University, 355 Luding Road, Shanghai, 200062 China; 3 Department of Molecular Pathology, The Affiliated Cancer Hospital of Zhengzhou University, Henan Cancer Hospital, Zhengzhou, 450008 Henan China; 4 Bio-ID Center, School of Biomedical Engineering, Shanghai Jiao Tong University, Shanghai, 200240 China; 5 Cancer Institute, Longhua Hospital, Shanghai University of Traditional Chinese Medicine, Shanghai, 200032 China

**Keywords:** Puberty, Hypothalamus, Spatial transcriptome, ARC, Kiss1, Slc18a3

## Abstract

**Background:**

A variety of neurons in hypothalamus undergo a complicated regulation on transcription activity of multiple genes for hypothalamic–pituitary–gonadal axis activation during pubertal development. Identification of puberty-associated cell composition and characterization of the unique transcriptional signatures across different cells are beneficial to isolation of specific neurons and advanced understanding of their functions.

**Methods:**

The hypothalamus of female Sprague–Dawley rats in postnatal day-25, 35 and 45 were used to define the dynamic spatial atlas of gene expression in the arcuate nucleus (ARC) by 10× Genomics Visium platform. A surface protein expressed selectively by kisspeptin neurons was used to sort neurons by flow cytometric assay in vitro*.* The transcriptome of the isolated cells was examined using Smart sequencing.

**Results:**

Four subclusters of neurons with similar gene expression signatures in ARC were identified. Only one subcluster showed the robust expression of Kiss1, which could be isolated by a unique membrane surface biomarker Solute carrier family 18 member A3 (SLC18A3). Moreover, genes in different subclusters presenting three expression modules distinctly functioned in each pubertal stage. Different types of cells representing distinct functions on glial or neuron differentiation, hormone secretion as well as estradiol response precisely affect and coordinate with each other, resulting in a complicated regulatory network for hypothalamic–pituitary–gonadal axis initiation and modulation.

**Conclusion:**

Our data revealed a comprehensive transcriptomic overview of ARC within different pubertal stages, which could serve as a valuable resource for the study of puberty and sexual development disorders.

**Supplementary Information:**

The online version contains supplementary material available at 10.1186/s13578-022-00745-2.

## Background

Adolescent sexual maturation is an important part of individual ontogeny in mammalian development. The active and high hormone levels in this juvenile period promote the fertility attainment, body growth, increased metabolism and acceleration of psychological development. The initiation of puberty is attributed to the increased gonadotropin-releasing hormone (GnRH) secretion in pulsatile manner and the subsequent activation of hypothalamic–pituitary–gonadal axis [1]. Kisspeptin encoded by KiSS-1 metastasis suppressor (*Kiss1*) gene has been proposed as a key regulator of puberty onset in bridging steroid levels and feedback effects on GnRH secretion for reproductive function [2]. Genetic etiology of *Kiss1* and its receptor *GPR54* genes is associated with hypogonadotropic hypogonadism and central precocious puberty [3].

Kiss1 neurons have been mapped within the arcuate nucleus (ARC) and anteroventral periventricular nucleus (AVPV) with dense continuum of cells as well as the dorsomedial nucleus and posterior hypothalamus with low-density group of scattered cells [4]. Besides that, the projection patterns of various Kiss1 neuron populations are also different. Kiss1 neurons at preoptic area project to the cell body and proximal dendrites of GnRH neurons and are responsible for the luteinizing hormone (LH) surge required for ovulation, while Kiss1 neurons in the ARC project to the distal dendron of GnRH neurons and generate the release of pulsatile GnRH [5]. Recently, researches have reported the extensive colocalization of neurokinin B (*NKB*) (encoded by *TAC3*) and dynorphin (encoded by *Pdyn*) with *Kiss1* expression. Therefore, Kiss1 neurons were also identified as kisspeptin/neurokinin B/dynorphin (KNDy) neurons[6]. Additionally, the coordination of neurotransmitters secreted from other glutamatergic and GABAergic neurons also seems to be strongly interconnected and synchronised with Kiss1 neurons around puberty [7, 8].

A fundamental step in understanding the neuroendocrine regulation of the reproductive axis requires accurate mapping of synaptic connectivity to KNDy neurons. Given the current research progress of the transgenic Kiss1 animal models, the transgenic GFP or β-galactosidase were too weak to be observed [9]. Moreover, the CRE-activated reporter usually drives the expression of transgenic target at the ectopic sites where kisspeptin protein is not regularly expressed [10]. The accurate characterization and isolation of KNDy neurons or other important neurons in vivo for molecular biological experiments (especially the next generation sequencing) was still missing.

Our previous studies have determined that the rats with postnatal day (PND)-25, 35 and 45 represent childhood, adolescent and adult stages via the fertile phenotypes as well as the dynamic changes of *Kiss1* and *GnRH* expression [11, 12]. Barcoding-based spatial transcriptomic sequencing [13] that provides the visualization of high-quality RNA-sequencing data in tissue with two-dimensional positional information gives us a clue for advanced understanding of the distribution and dynamic changes of cell-specific neurons in wild type rodents during pubertal process without any transgenic symbols. In this study, we used 10× Genomics Visium platform to generate transcriptome-scale spatial gene expression in ARC regions of three individual female Sprague–Dawley (SD) rats with the different ages of PND-25, 35 and 45. By comparison among the different hypothalamic cell clusters, our data uncovered important novel hallmarks for specific Kiss1 neurons isolation. We provide these data as a significant resource for the neuroscience community to augment current molecular profiling and spatial transcriptomics efforts in the neuroendocrinology for pubertal development.

## Materials and methods

### Experimental animals

Female Sprague–Dawley rats in PND-25, 35 and 45 purchased from Shanghai SLAC Laboratory Animal Co., Ltd. (Shanghai, China) were allowed free access to food and water. Rats were sacrificed via cervical dislocation. The whole brains and ovaries were isolated immediately. Ovaries were sectioned by 5 μm and conducted for hematoxylin–eosin (HE) staining (C0105S, Beyotime, Shanghai, China). The area of active corpus luteum was assessed for the sex maturation. Ten rats in each pubertal stage were collected for flow cytometric assay. All the procedures were followed by the Institutional Animal Care and Use Committee of Shanghai Children’s Hospital.

### Spatial transcriptomic sequencing

OCT-embedded whole brains were cryosectioned coronally to a thickness of 10 μm (bregma: − 2.52 to − 2.92, interaural: 6.08 to 6.48 mm) using a CryoStar NX70 cryostat (Thermo Fisher Scientific, Waltham, MA, USA). Tissue sections were layered onto the visium spatial tissue optimization slide containing oligonucleotides for mRNA capture (10× Genomics, Pleasanton, CA, USA). Each capture area per slide has 5000 spatially barcoded with a diameter of 55 μm and a center-to-center distance of 100 μm, over an area of 6.5 mm by 6.5 mm. A Master Mix containing reverse transcription (RT) reagents and fluorescently labeled nucleotides is added on top of the tissue sections, resulting in fluorescently labeled cDNA synthesis. Tissue is enzymatically removed, leaving behind fluorescent cDNA covalently linked to oligonucleotides on the slide. Fluorescent cDNA is visualized under fluorescence imaging conditions verified using the Visium Imaging Test Slide. Fluorescent cDNA is visualized under fluorescence imaging conditions verified using the Visium Imaging Test Slide. H&E and fluorescence images are compared. The permeabilization time that results in maximum fluorescence signal with the lowest signal diffusion is optimal. The libraries were sequenced with paired-end 150 bp sequencing (PE150) by NovaSeq 6000 platform.

Space ranger showed the capture area of the tissue in the slide and differentiates reads for each spot based on spatial barcode information. STAR was used to assess the sample quality by total number of spots, the number of pairs of reads in each Spot, the number of detected genes, and the number of unique molecular identifier (UMIs). Sctransform was used to normalize data, construct regularized negative binomial model of gene expression, and detect high variance characteristics. The method of t-distributed stochastic neighbor embedding (t-SNE) and uniform manifold approximation and projection for dimension reduction (UMAP) was used for dimension reduction. K-means was used to cluster the same type spots together. Seurat was used to identify the genes with spatial expression tendency through unsupervised clustering or prior knowledge. SPOTlight was used to identify the cell types based on deconvolution algorithm for nonnegative matrix factorization. MAST difference test method was used to evaluate differential expressed genes (DEGs) under adjusted *p*-values < 0.001. Gene ontology (GO) was used to analyze the functions and associated signaling pathways of DEGs.

### Flow cytometric (FACS) assay

Fresh isolated ARC tissues from SD rats with PND-25, 35 and 45 (n = 10 each group) as previously described [14] were washed by 1 × PBS, and processed using Neural Tissue Dissociation Kits (Cat. No. 130-092-628, Miltenyi Biotec, Koln, Germany) and harvested single cells by Gentle MACS™ Octo Dissociator With Heaters (Miltenyi Biotec). 5 × 10^7^ Cells were suspended into 5 × 10^6^/ml density by cold PBS containing with 1% sodium azide, and incubated with 20 μl solute carrier family 18 member A3 (SLC18A3) antibody (Cat. No. A16068, Abclonal, Wuhan, Hubei, China) for 30 min at 4 °C. After washing by PBS for three times, 10 μl secondary antibody of goat anti rabbit IgG H&L conjugated with FITC (Cat. No. ab6717, Abcam, Cambridge, MA, USA) was added to incubate for 30 min at 4 °C in dark. Cells were washed by PBS for another three times, and resuspended into cold PBS containing with 3% BSA and 1% sodium azide, followed by 5 μl 7-AAD (Yeasen, Shanghai, China) incubation for 15 min in dark.

Cells were analyzed on FACSCanto II (BD Bioscience, Franklin Lakes, NJ, USA) and screened on AriaIII (BD Bioscience). The following gating strategy was adopted to obtain SLC18A3 positive cells: first, FSC A/H gating was used to remove adherent cells, and then 7-AAD positive were removed to exclude dead cells. After that, FSA/SSA gating was used to remove fragments, and two cell population were obtained, namely FSA large or small cell. Finally, four groups of cells were sorted to obtain SLC18A3-positive large cells, SLC18A3-positive small cells, SLC18A3-negative large cells, SLC18A3-negative small cells.

### Smart sequencing

Cells harvested from FACS were centrifuged at 10,000*g* for sheath fluid removal, and suspended by 10 μl lysis buffer (Cat. No. 635013, ClonTech Laboratories, Mountain View, CA, USA) containing with 40 U Recombinant Rnase Inhibitor (Cat. No. 2313Q, ClonTech Laboratories). RNA was then processed for reverse transcription using SMARTer Ultra Low Input RNA Kit for Sequencing v3 (Cat. No. 634852, ClonTech Laboratories). The libraries were constructed using KAPA Hyper Prep Kit (Cat. No. KK8504, Roche, Basel, Switzerland), and sequenced with PE150 by NovaSeq 6000 platform.

The raw data was trimmed adaptors and filter out low quality reads using Trimmomatic (non-default parameters: SLIDINGWINDOW:4:15 LEADING:10 TRAILING:10 MINLEN:35), and checked the quality of clean reads using Fastqc. Next, clean reads were aligned to the latest mouse genome assembly mm10 using Hisat2 v2.0.5 (non-default parameters: –rna-strandness RF –dta). The transcripts were assembled and the expression levels were estimated with FPKM values using the StringTie algorithm (non-default parameters: –rf). Differential mRNA and lncRNA expression among the groups were evaluated using an R package Ballgown, and the significance of differences (log_2_|FC|> 1, *q* value < 0.05) by the Benjamini & Hochberg (BH) adjustment method were computed. Gene annotation was described by Ensembl genome browser database (http://www.ensembl.org/index.html). The R package ClusterProfiler was used to annotate the differential genes with GO analysis.

### Immunofluorescence (IF) assay

The freshly prepared frozen 20 μm sections were placed in 1 × PBS and rinse three times for 10 min each time, and then transfered the slices to the mounting solution (1 × PBS 18.4 ml, BSA 0.2 g, 10% TritonX-100 800 μl, goat serum 800 μl). Primary antibody diluent (0.3% TritonX-100 75 μl, 1 × PBS 24.75 ml, goat serum 250 μl) and secondary antibody diluent (0.3% TritonX-100 250 μl, 1 × PBS 25 ml) were prepared. After sealing at 37 °C for 1 h, the brain tissue slices were placed in a 200 μl tube and transferred to a 4 °C refrigerator for primary antibodies (Kisspeptin, 1:500, Cat. No. AB9754, Millipore, Billerica, MA, USA; NR5A2, 1:100, Cat. No. NBP2-46248, Novus, Centennial, CO, USA; TGFBI, 1:50, Cat. No. NBP1-47215, Novus; SPP1, 1:100, Cat. No. NB100-1883, Novus; SLC18A3, 1:100, Abclonal) incubation overnight. The next day, the sections were rinsed three times with 1 × PBS for 10 min each time. All subsequent operations were carried out in the dark. The brain tissue section were replaced again in a new 200 μl tube and incubate with the secondary antibodies (donkey anti goat, 1:50, Cat. No. SA00003-3, Proteintech Group, Rosemont, IL, USA; donkey anti rabbit, 1:300, Cat. No. SA00013-8, Proteintech Group; donkey anti mouse, 1:100, Cat. No. SA00003-9, Proteintech Group) at room temperature for 1 h. After washing by 1 × PBS for six times, the brain slice were placed on an adhesive glass slide, and placed it in a light-proof humidifier to dry it to avoid peeling off during the subsequent process. The sections were covered the sections with DAPI (20 μl), and removed the DAPI and absorbed the water around the tissue after 20 min of the nuclei staining, then added anti-fluorescence quenching mounting tablets (10–20 μl/sheet) to each tissue, and mounted the slides with a cover glass.

### Data deposits

The raw sequencing data was deposited to ArrayExpress assigned with the accession number E-MTAB-10975 and E-MTAB-10976.

## Results

### Overview of hypothalamic cell clusters identified by spatial transcriptomic sequencing

**Fig. 1:**
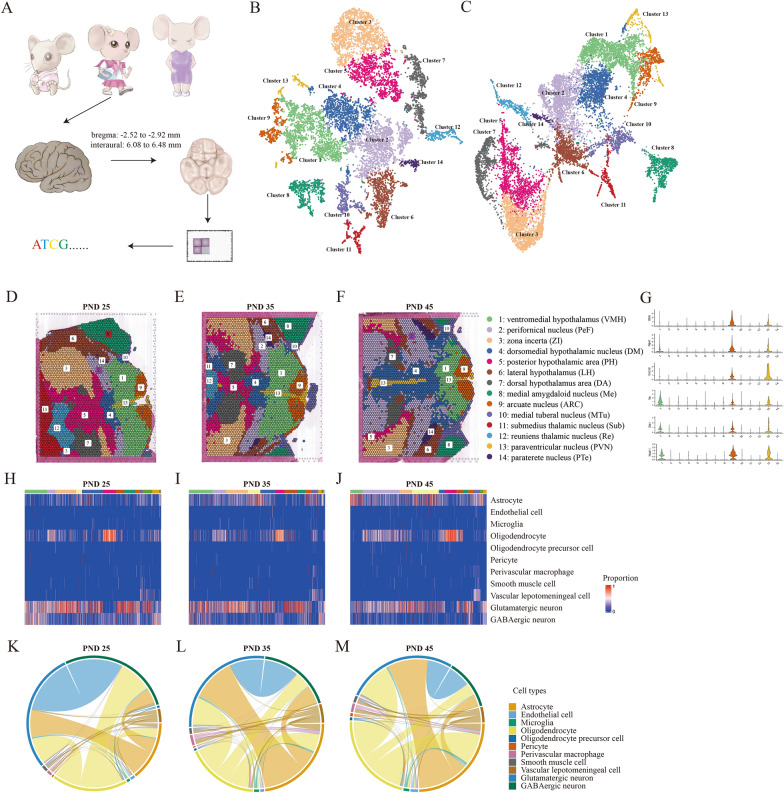
Cell clusters identification in rat hypothalamus by spatial transcriptomics sequencing. **A** Schematic diagram of spatial transcriptomics sequencing of rat hypothalamus with postnatal day (PND)-25, 35 and 45. The target hypothalamus is sectioned at bregma: − 2.52 to − 2.92 mm and interaural: 6.08 to 6.48 mm. The tSNE (**B**) and UMAP (**C**) plots showing the cells are classified into 14 clusters based on the transcriptomes of overall gene expression relationship among the 12,436 spots with 5179 genes. Different cell clusters are color-coded. The clusters of ventromedial hypothalamus nucleus (VMH) (Cluster 1), arcuate nucleus (ARC) (Cluster 9), and paraventricular nucleus (PVN) (Cluster 13) are highlighted. **D**–**F** Feature plots showing the distribution of clusters in brain sections of PND-25, 35 and 45. The colors and names of each cluster are corresponding to **B**. **G** Violin plots showing the gene expressions of *Ghrh*, *Trh*, *Npvf*, *Dlk1*, *Ces1d* and *Nkx2-1* in all 14 clusters. **H**–**J** Cell type heatmap showing the proportion of different cell types occupied in each cluster of PND-25, 35 and 45. **K**–**M** Chord diagrams showing that all spot sites on a spatial slice of a sample contain both specific cells of PND-25, 35 and 45. The thicker lines represent that these two cell types share more spots

Female SD rats aged PND-25, 35 and 45 were examined for the ovarian maturation. The area of active corpus luteum (≥ 30 mm) occupied in ovarian structure indicated the different reproductive ability during these pubertal stages (Additional file 1: Fig. S1). The whole brains were performed 10 μm serial tissue sections transversely to expose ARC regions (bregma: − 2.52 to − 2.92 mm, interaural: 6.08 to 6.48 mm) according to Allen Brain Atlas [15] and processed by spatial transcriptomic sequencing (Fig. 1A). The total 12,436 spots containing 10 cells average were sequenced to a median depth of 497.9 M reads with 5179 genes per spot and 29,277 UMIs (Additional file 2: Table S1). Quality control for the ratio of mitochondrial gene counts, expressed gene counts and unique molecular identifiers in each spot ensure the qualified sequencing data of tissues without cellular apoptosis or cytolysis (Additional file 1: Fig. S2A–C).

Fourteen cell clusters in the detected regions of integrating three samples have been characterized by t-SNE (Fig. 1B) and UMAP (Fig. 1C) dimension reduction clustering analysis. We observed a series of clear-cut neurons in hypothalamus and adjacent regions distinguished by the global differentially expressed signatures in spots (Fig. 1D–F), and the areas, structures and positions of all these neurons were generally similar in three samples. We observed that the clusters of ARC (Cluster 9), ventromedial hypothalamus (VMH) (Cluster 1) and paraventricular nucleus (PVN) (Cluster 13) gathered closely to each other compared to other clusters, indicating that the nucleus distributed in the adjacent regions displayed more similar gene expression profiles. Given the hallmarks of each cell type (Additional file 3: Table S2), we noticed that many specific marker genes such as *Ghrh*, *Npvf* and *Ces1d* in ARC as well as *Trh*, *Dlk1* and *Nkx2-1* in VMH were also co-expressed in PVN (Fig. 1G, Additional file 1: Fig. S3), which suggested that genes exhibited a gradient expression profiles across the transition zones among adjacent neuronal clusters. Moreover, these fourteen neuronal clusters could be further divided into GABAergic and glutamatergic neuronal cells, and nine non-neuronal cell types (Fig. 1H–J, Additional file 1: Fig. S4) by the combined analysis of the resources of single cell sequencing data of rat brain from “Allen Brain Atlases and Data” (https://portal.brain-map.org/) [16]. From the perspective of the interplay of different cell types made up in each spot in spatial transcriptomic sequencing, the overall change of proportions across different pubertal stages was obvious. For example, the occurrence of glutamatergic and GABAergic neurons as well as GABAergic neurons and oligodendrocytes were gradually decreased from PND25 to PND45. In turn, the occurrence of glutamatergic neurons and astrocytes, glutamatergic neurons and oligodendrocytes, as well as astrocytes and oligodendrocytes were increased (Fig. 1K–M). Our initial glance indicated that ARC, PVN, VMH and other hypothalamic regions closely associated with pubertal development were all shown in our spatial transcriptomics data.

### Identification and characterization of KNDy neurons in rat hypothalamus

**Fig. 2:**
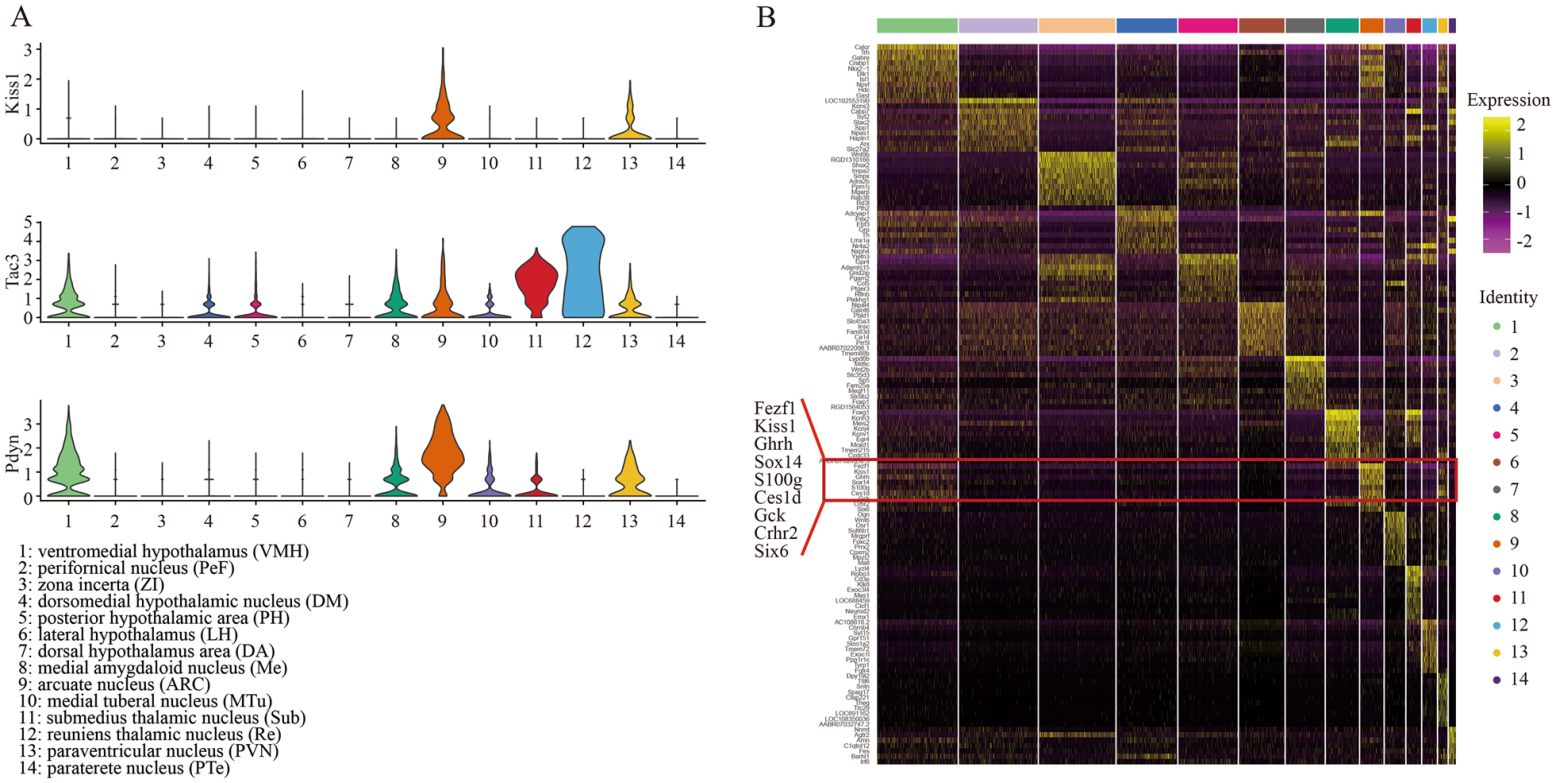
Spatial transcriptomic profiles of ARC. **A** Violin plots showing the gene expressions of *Kiss1*, *Tac3* and *Pdyn* in all 14 clusters. **B** Heatmap showing the top ten genes in 14 clusters. Red frame highlights the top expressed genes in ARC (Cluster 9)

Due to the crucial roles of KNDy neurons in puberty initiation, we majorly focused on the expressions of KNDy (*Kiss1*, *Tac3* and *Pdyn*) in whole hypothalamic regions. The *Kiss1* positive spots were concentrated on ARC, PVN and scattered in other regions, whereas *Tac3* and *Pdyn* were robustly enriched not only in the neurons above, but also in VMH (Cluster 1), dorsomedial hypothalamic nucleus, dorsal part (DMD) (Cluster 4), posterior hypothalamic area (PH) (Cluster 5), medial tuberal nucleus (MTu) (Cluster 8), VMH ventrolateral part (VMHVL)(Cluster 10), ventral reuniens thalamic nucleus (VRe) (Cluster 11) and reuniens thalamic nucleus (Re) (Cluster 12) (Fig. 2A, Additional file 1: Fig. S5). The widely acknowledged functions of NKB and dynorphin on mental controlling, pain repression and addiction indicated that these two peptides extensively existed in multiple types of neurons and were not the appropriate biomarkers for KNDy neurons tracing and isolation. Top ten biomarkers (*Fezf1*, *Kiss1*, *Ghrh*, *Sox14*, *Npvf*, *S100g*, *Ces1d*, *Gck*, *Crhr2*, *Six6*) of Cluster 9 distinguishing ARC from other thirteen clusters were provided to address whether there were any other hallmarks being used for KNDy neurons identification besides *Kiss1* (Fig. 2B), and we observed that *Sox14*, *S100g*, *Gck* and *Six6* were uniquely expressed in Cluster 9 more specific than *Kiss1* (Additional file 1: Fig. S6).

**Fig. 3:**
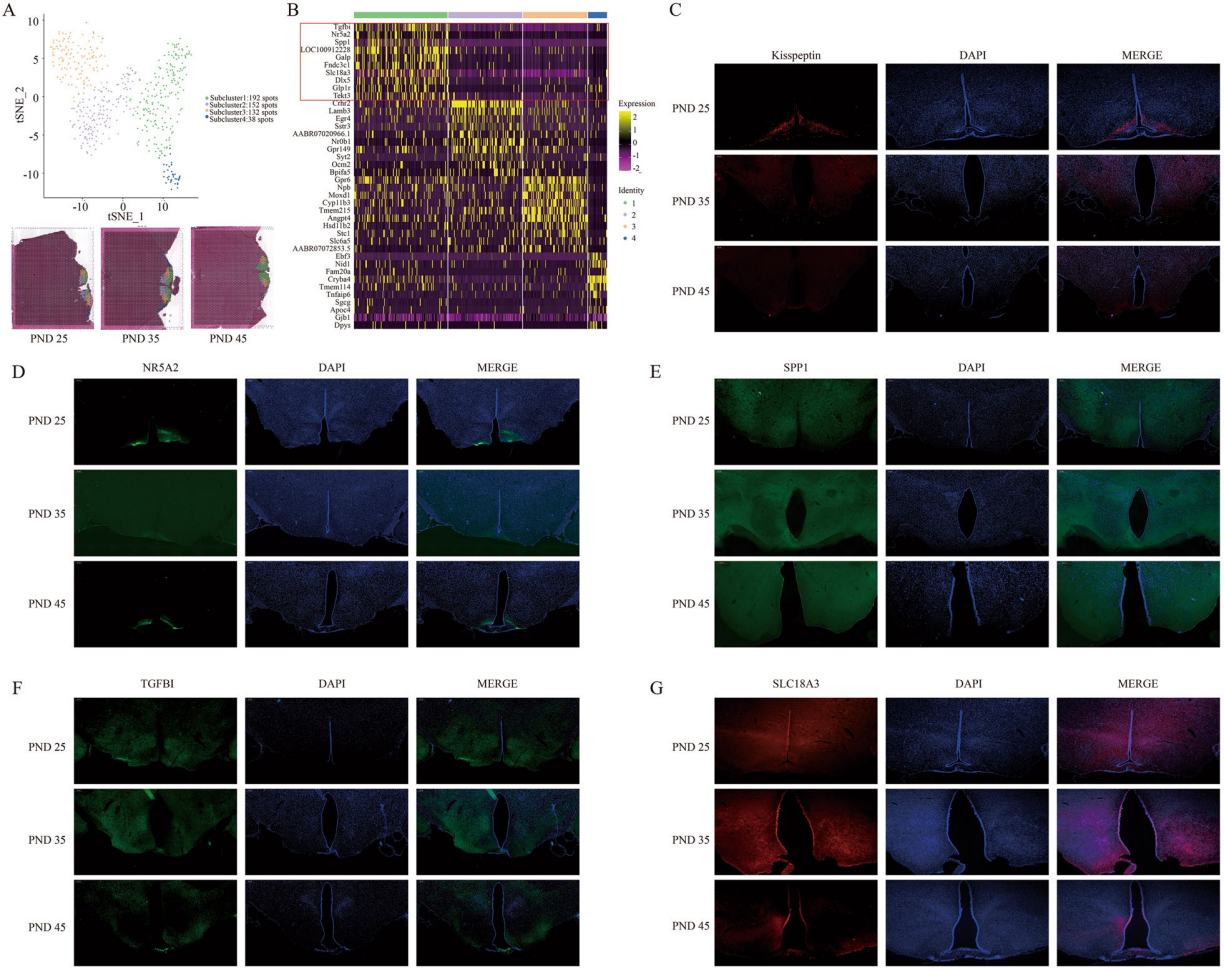
Cell clusters identification in ARC. **A** The tSNE plot showing the cells are classified into 4 clusters based on the transcriptomes of overall gene expression in 514 spots of ARC (Cluster 9) (Top). Feature plots showing the distribution of clusters in PND-25, 35 and 45 (Bottom). **B** Heatmap showing the top ten genes in these 4 subclusters. Red frame highlights the top expressed genes in Subcluster 1. **C**–**G** The IF assay showing the expression of *Kiss1*, *Nr5a2*, *Spp1*, *Tgfbi* and *Slc18a3* in ARC of PND-25, 35 and 45 rat

Next, ARC containing abundant KNDy positive neurons was further divided into more subclusters according to the comprehensive gene expression. Four subtypes of neurons in ARC were shown (Fig. 3A). Herein, *Kiss1* (FC = 1.417, *p* = 3.58 × 10^–24^) and *Tac3* (FC = 2.563, *p* = 5.02 × 10^–72^) were highly expressed in the Subcluster 1 of Cluster 9 compared to other three subclusters, while *Pdyn* was highly expressed in Subcluster 2 (FC = 1.065, *p* = 5.12 × 10^–59^) (Additional file 4: Table S3), indicating that population in Subcluster 1 was likely to contain a great deal of Kiss1*-*expressed neurons. KNDy neurons might be not equal with Kiss1-expressed neurons because of the differential localization of *Pdyn* expression. In Cluster 9, top ten genes of *Tgfbi*, *Nr5a2*, *Spp1*, *Galp*, *Fndc3c1*, *Slc18a3*, *Dlx5*, *Glp1r*, *Tekt3*, and *Kiss1* could be considered as the appropriated candidate hallmarks to distinguish the largest number of *Kiss1* positive neurons in Subcluster 1 from other three subclusters (Fig. 3B). *Tgfbi*, *Nr5a2*, *Spp1* and *Slc18a3* were picked up to examine their expression compared to *Kiss1* in ARC by IF (Fig. 3C–G), the specificity of *Nr5a2* and *Slc18a3* in ARC beyond other regions was observed. In our data, PVN had too few spots to perform subclustering analysis, and although VMH could be also divided into four subclusters (Additional file 1: Fig. S7), the expression of *Kiss1* (no statistical significance), *Tac3* (FC = 1.269, *p* = 4.41 × 10^–57^ in Subcluster 3) and *Pdyn* (FC = 1.3, *p* = 1.03 × 10^–68^ in Subcluster 4) (Additional file 5: Table S4) implied that KNDy neurons were seemingly scattered but not centrally distributed in VMH. Taken together, the spatial transcriptomics sequencing data provided applicable biomarkers for KNDy neurons characterization from wild type rat hypothalamus.

### Kiss1-expressed neuron isolation by Slc18a3

**Fig. 4:**
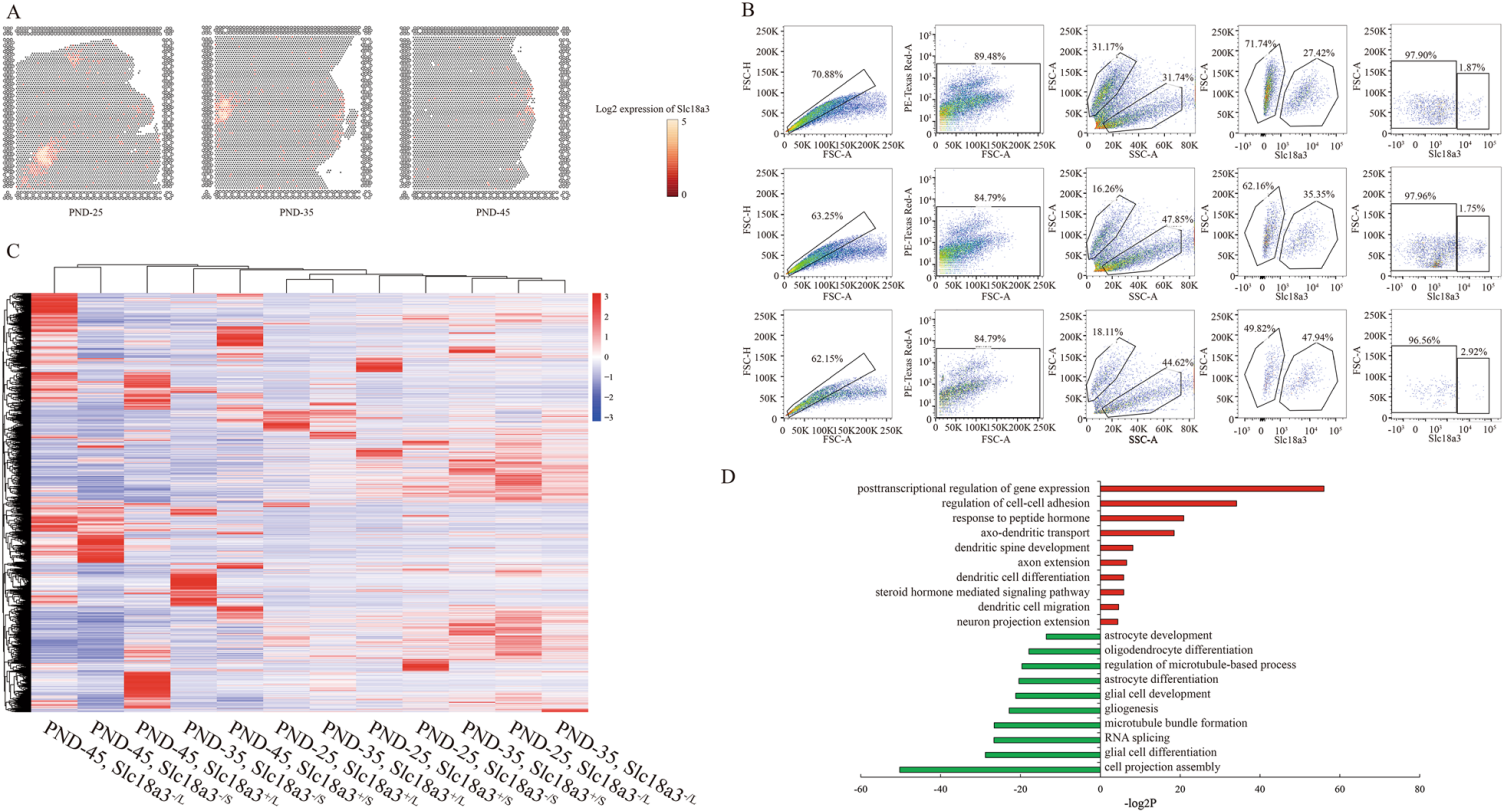
Isolation of *Kiss1*-expressed cells from ARC. **A** Feature plots showing the *Slc18a3*-expressed spots in PND-25, 35 and 45. **B** Flow cytometric analysis showing the process of ARC cells isolated by SLC18A3 from PND-25 (Top), PND-35 (Middle) and PND-45 (Bottom). **C** Heatmap showing the gene expression signatures of the four populations from **B** in PND-25, 35 and 45. “L”: large; “S”: small; “+”:SLC18A3 positive; “−”: SLC18A3 negative. **D** GO analysis of differential expressed genes (DEGs) between the large and small SLC18A3 positive cells. Red items represent the up-regulated DEGs while green items represent the down-regulated DEGs in large cells compared to the small ones

Kisspeptin, as a secreted protein, is not suitable for KNDy neuron isolation despite the best expression specificity. In top ten DEGs in Cluster 9, the cytomembrane surface expressed marker *Slc18a3* was highly expressed in ARC, and central medial thalamic nucleus (Cluster 12 in Fig. 1C–E) (Fig. 4A) was used to examine the practicability of alive Kiss1-expressed neuron isolation from the hypothalamus by flow cytometric assay. Here, we obtained two populations according to the cell size by FSC/SSC signals, and further harvested a small proportion of Slc18a3^+^ cells from hypothalamus of PND-25, 35 and 45 (Fig. 4B). Slc18a3^+^ large cell counts seemed to gradually increase from PND-25 to PND-45 (Fig. 4B). To verify the effectiveness of this methods for isolation of living neurons with high expression of *Kiss1*, smart-seq was conducted to detect the transcriptome of four population namely large Slc18a3^±^ cells as well as small Slc18a3^±^ cells. The transcriptome of large Slc18a3^+^ cells displayed highest expression of *Kiss1*, *Tac3* and *Pdyn* compared to other three population (Fig. 4C, Additional file 6: Table S5). Concurrently, candidate genes including *Nr5a2*, *Tgfbi* and *Slc18a3* were highly expressed in both large and small Slc18a3^+^ cells. Interestingly, *Gfap*, *Aldh1l1*, *Sox10*, and *Olig2* were robustly expressed in small Slc18a3^+^ cells compared to large ones. Combined with the cell sizes, we speculated that the large ones were probably neurons while small ones were glial cells. As expected, GO analysis on DEGs compared between large and small Slc18a3^+^ cells showed that large population presented function on axon and dendrites development as well as hormone transport, whereas small population presented glial differentiation (Fig. 4D). Taken together, we provided a reliable method to isolate living Kiss1-expressed neurons from hypothalamus of wild type rats.

### Dynamic changes of gene expression in ARC in different stages of puberty

**Fig. 5:**
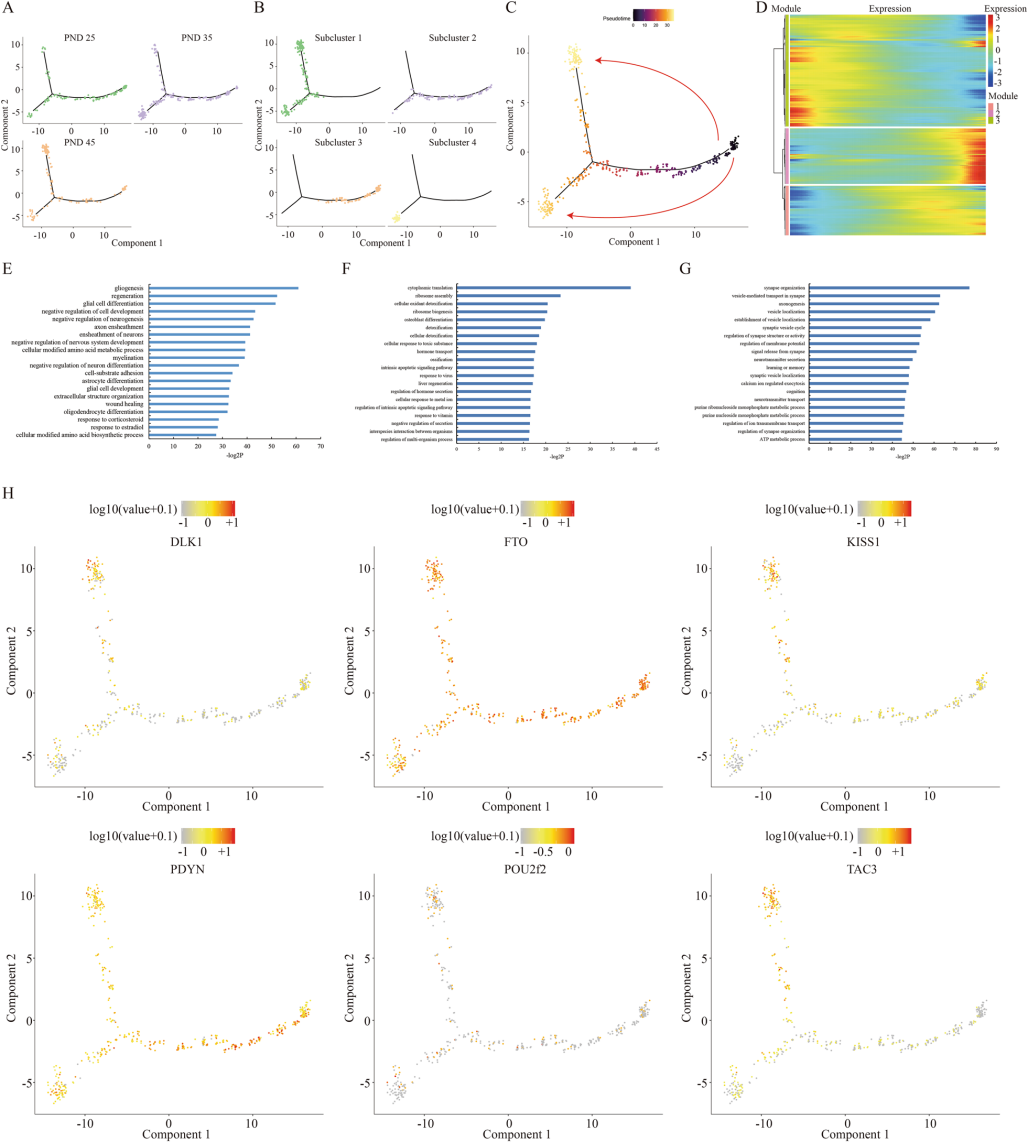
Transcriptional dynamics during pubertal development. **A** Unsupervised pseudotime analysis showing the PND-25 (green), 35 (pinkish purple) and 45 (orange) based on their gene expression profiles. Minimal spanning tree is shown in black. **B** Unsupervised pseudotime analysis showing the Subcluster 1 (green), Subcluster 2 (pinkish purple), Subcluster 3 (orange) and Subcluster 4 (yellow) based on their gene expression profiles. **C** Unsupervised ordering of the pubertal development based on gene expression profiles of these three stages. Arrows indicate the direction of development. **D** Heatmap showing three groups of gene expression modules with distinct expression dynamics during pubertal development. **E**–**G** GO analysis of the genes in three modules, respectively. **H** Scatterplots showing the expression of *Dlk1*, *Fto*, *Kiss1*, *Pdyn*, *Pou2f2* and *Tac3* along the pseudo-timeline

Although kisspeptin was tightly connected with puberty, the precise and clear dynamics of gene expression profile of Kiss1-expressed neurons in hypothalamus had never been described yet. The spatial transcriptomic sequencing of ARC at different pubertal stages provided an opportunity for understanding the transcriptional program of Kiss1-expressed neurons in vivo*.* To this end, an unsupervised pseudotime analysis was conducted in four subclusters of ARC. We noticed that the spots of ARC in PND-45 (Fig. 5A) were largely in accordance with the spots of Subcluster 1 (Fig. 5B), whereas the spots of ARC in PND-25 majorly overlapped with the spots of Subcluster 3, and the spots of ARC in PND-35 were involved in all subclusters, suggesting that the pseudotime axis of cell programming well mimicked the pubertal development (Fig. 5C, Additional file 1: Fig. S8). Nevertheless, it was noteworthy that the overall transcriptome of a proportion of ARC neurons in PND-35 were different from in PND-45 (Fig. 5C), indicating two parallel developmental routes of ARC neurons from PND-25 to PND-35 as well as from PND-25 to PND-45, but not a one-way route from PND-25, PND-35 to PND-45. In other words, the distinct subclusters between PND-35 and PND-45 might reflect the differences of genotype between puberty and complete sex maturation.

Moreover, three modules with the distinct expression landscape of 2,948 hypervariable genes in each module were characterized along the puberty process based on the gene expression tendency (Fig. 5D, Additional file 7: Table S6). Given the related biological processes among different modules of gene expression by GO analysis, genes in Module 1 contributed to enhancing glial cells but repressing neuron cells proliferation induced by estradiol (Fig. 5E), and genes in Module 3 were responsible for neurons differentiation and signals transmission (Fig. 5G). Interestingly, only genes in Module 2 substantially played a regulatory role in hormone secretion (Fig. 5F). Correspondingly, we exemplified the expression of *Pdyn* and *Fto* (Module 3) as well as *Kiss1*, *Tac3*, *Dlk1* and *Pou2f2* (Module 2) in different modules by pseudotime analysis (Fig. 5H), indicating that multiple genes in ARC participated in pubertal onset and development actually via distinct functions.

### Neuron type alteration across puberty

**Fig. 6:**
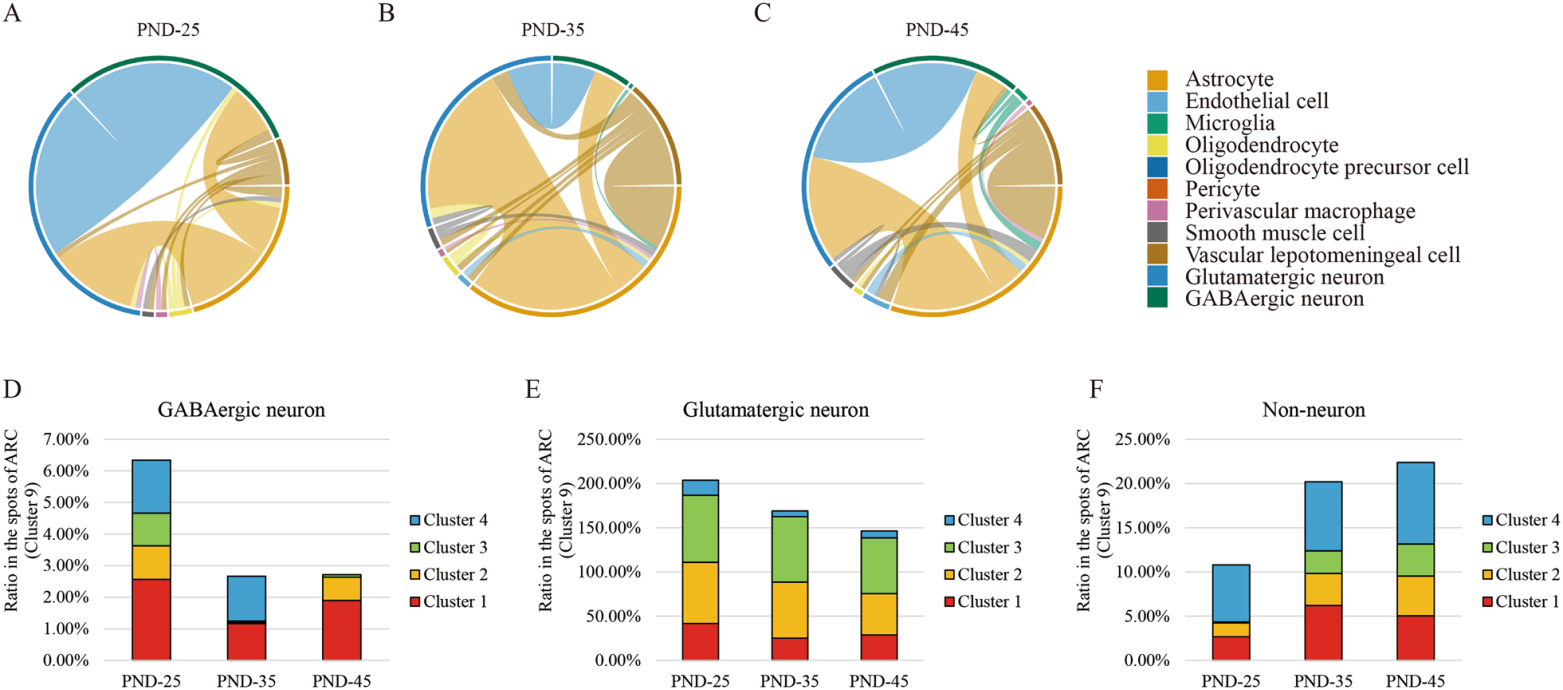
Cell types alteration during pubertal development. **A**–**C** Chord diagrams showing the intercrossed cell types in PND-25, 35 and 45. The thicker lines represent that these two cell types share more spots. **D**–**F** The proportion dynamics of GABAergic, glutamatergic and non-neuron in four subclusters in PND-25, 35 and 45

In ARC, our clustering analysis identified a number of GABAergic neuron subtypes, glutamatergic neuron subtypes, and non-neuronal subtypes, which were intercrossed with each other (Fig. 6A–C). In the annotated neuron types, GABAergic neurons with high expression of Meis2, glutamatergic neurons as well as astrocytes were largely coordinated with a variety of other cells throughout the entire route of sexual development. The components of these neuron mixture dynamically changed across the puberty (Fig. 6D–F, Additional file 1: Fig. S9). We found that GABAergic and glutamatergic neurons were both gradually replaced by non-neuron cells appearing in these four clusters from PND-25 to PND-45. Collectively, these results demonstrated that our unbiased data analysis were able to reveal the particular cell types in ARC that contributed to puberty initiation and development.

## Discussion

The current study provides the spatial transcription atlas of multiple neuronal nuclei within ARC across different stages of puberty. Owing to the limitation of spots in our spatial transcriptome data, many specific neurons such as *Pomc-*, *Ghrh-* and *Agrp*-highly-expressed neurons are all classified as Subcluster 1, which displays a relatively rough description on the coordination of neurons in ARC compared to single-cell RNA-seq. Additionally, the data of AVPV as the other main group of *Kiss1*-expressed neuron bodies, median eminence as well as ARC in sexual development related disease model such as central precocious puberty is not included in this study. Nevertheless, compared to previous single-cell sequence studies on hypothalamus [17–19], our data uniquely extend the dynamic spatial identity of neurons nucleus subsets within ARC specifically for the event of female mammalian puberty. Our findings will be an abundant source for evaluation of transcriptional profiles in central precious puberty and sexual dysplasia, and provide comparative neuroendocrine data of for future studies.

Previous studies on single-cell RNA-seq have determined that ARC can be divided into eleven clusters in embryonic E15 brain [20], and twenty clusters in adult brain [21] by gene expression signatures. The signaling of kisspeptin-GPR54 at GnRH neurons are critical for maintaining GnRH release needed for fertility especially in puberty. In general, kisspeptin-expressed neurons are majorly located in the rostral periventricular area of the third ventricle of rodents or the preoptic area of other mammals and the ARC of the hypothalamusare sites of the highest density of kisspeptin expression within the mammalian brain [6]. Due to the high degree of colocalization of the other two peptides (Neurokinin B and Dynorphin) with kisspeptin, and for simplicity, this population of kisspeptin-expressed neurons is also abbreviated as KNDy neurons. Although more detailed and comprehensive information of neurons in ARC have been characterized, the spatial position of KNDy neurons are not fully elucidated in ARC in pubertal event. Our data fills this gap to some extent, and provides the distribution and the alteration of these four clusters in ARC during pubertal development (Fig. 3A). From our results, we notice that the cell clusters with the highest expression of *Pdyn* are not identical with *Kiss1* and *Tac3* in ARC (Additional file 4: Table S3), suggesting that *Pdyn* may play a more extensive role in neuroendocrine beyond the reproductive function, and seem to be not a specific biomarker for KNDy neurons.

In turn, the top ten highly expressed genes in Subcluster 1 indicate a series of potential hallmarks for Kiss1 neurons. For example, transforming growth factor beta induced (*Tgfbi*) is never reported in puberty, and first discovered in Subcluster 1 of ARC. TGFBI exerted as a tumor suppressor or enhancer in cancer progression is an extracellular matrix (ECM) protein that is associated with a number of ECM proteins such as fibronectin, biglycan, decorin, and several types of collagen and functions as a ligand to modulate cell adhesion and migration via various types of integrins [22]. The high-expressed *Tgfbi* is supposed to be of benefit to the microenvironment for the signals communication among different neurons in ARC. Another important example is nuclear receptor subfamily 5 group A member 2 (*Nr5a2*), a critical regulator for adult neurogenesis [23]. *NR5A2* is sufficient to reduce cell proliferation, govern fate specification and differentiation of adult neural stem cells, and promote axon outgrowth. Moreover, Nr5a2 can bind to the promoter of *Kiss1* for stimulating *Kiss1* transcription particularly in Kiss1 neurons of ARC but not AVPV [24] and regulates gonadotrope function [25], which suggests a molecular basis for the distinct regulation of basal kisspeptin expression between ARC and AVPV neurons.

In previous studies of the epigenetic mechanism on central puberty, specimens used for next generation sequencing or microarray are almost harvested from the entire ARC[26] and even hypothalamus tissue[27], which suggests an obscure landscape of gene expression profiles. Isolation of living Kiss1 neurons is necessary and benefit to culturing primary cell line, and carrying on more subsequent genetic experiments in vitro for puberty study. In our study, *Slc18a3* is first discovered and examined the specificity of Kiss1 neurons by flow cytometric assay. Although Slc18a3^+^ cells only account for a very small proportion of the whole ARC tissues, this population can be further divided according to the cell size and density of intracellular particles. The cell number is gradually increased from PND-25 to 45 in the large group, while is relatively stable in the small group (Fig. 4B). The gene profiles by smart-seq indicate that the Slc18a3^+^ large population shows the higher expression of *Kiss1*, *Nr5a2*, *Tgfbi*, and *Glp1r* compared to negative population, which is closer to Subcluster 1 than the Slc18a3^+^ small population. Although the small population has been found the robust expression of *Gfap*, *Aldh1l1*, *Sox10*, and *Olig2*, which are the classic biomarkers of astrocyte, microglia and oligodendrocyte, the phenotype and function of Slc18a3^+^ cells still need to be further explored before we can truly define them. Nevertheless, we are able to isolate the Kiss1-expressed cells with high purity from wild type rat by our methods. *Slc18a3* encodes for the vesicular acetylcholine transporter for loading newly synthesized acetylcholine from the neuronal cytoplasm into synaptic vesicles. It is a significant discovery that Kiss1 neurons have partial property of acetylcholinergic neurons, in which the role of *Slc18a3* in pubertal regulation or other function needs to be figured out in future research.

Finally, it is significant to investigate the alteration of gene signatures from a single-cell perspective during puberty. Three different modules of gene expression in four subclusters in ARC summarize the dynamic changes at the specific pubertal stages (Fig. 5C, D), indicating a novel rule of transcriptional regulation in ARC. For example, *Rbm38*, *Ptbp1*, *Mapkapk2*, *Ybx1*, *Samd4b*, *Carhsp1*, *Vim* and *Pabpc1* contributing to RNA stabilization and 5′ or 3′ UTR binding are all classified in Module 1, in which genes expression are increased by pseudotime analysis. The post-transcriptional regulation seems to be a new field to study puberty. These genes expressed in different types of cells representing distinct functions on glial or neuron differentiation, hormone secretion as well as estradiol response precisely coordinate and affect with each other, and result in a complicated regulatory network for hypothalamic–pituitary–gonadal axis initiation and modulation.

## Conclusion

In summary, our data revealed a comprehensive transcriptomic atlas of ARC with different pubertal stages, which could serve as a valuable resource for puberty and sexual development disorders study.

## Supplementary Information

**Additional file 1: Figure S1.** H&E staining of ovaries in PND-25, 35 and 45. The solid circular substances indicate corpus luteum. “*” and “**” represent the p-value less than 0.05 and 0.01 adjusted by Student’s t-test. **Figure S2.** Quality control of spatial transcriptomics sequencing. **Figure S3.** Top 10 highly-expressed genes in 14 clusters shown by violin plots. Refer to Fig. 2 for the name of 14 clusters. **Figure S4.** Different cell types shown by feature plots across PND-25, 35 and 45. **Figure S5.** Expression of Kiss1, Tac3, Pdyn and Slc18a3 shown by feature plots across PND-25, 35 and 45. **Figure S6.** The top 10 highly-expressed gene of Cluster 9 shown by violin plots in all 14 clusters. **Figure S7.** The tSNE plot showing the cells are classified into 4 subclusters based on the transcriptomes of overall gene expression relationship among the 1,787 spots of VMH (left). Feature plots showing the distribution of clusters in PND-25, 35 and 45 (right). Different cell clusters are color-coded. **Figure S8.** The dynamics of spots based on the gene expression profiles along the pseudo-timeline shown by feature plots. **Figure S9.** The proportion of different cell types in ARC of PND-25, 35 and 45 shown by feature plots.

**Additional file 2: Table S1.** The basic information of spatial transcriptomics data.

**Additional file 3: Table S2.** Information of DEGs with *p*-value less than 0.01 adjusted by MAST difference test method in all 14 clusters.

**Additional file 4: Table S3.** Information of DEGs with *p*-value less than 0.01 adjusted by MAST difference test method in 4 subclusters of ARC (Cluster 9).

**Additional file 5: Table S4.** Information of DEGs with *p*-value less than 0.01 adjusted by MAST difference test method in 4 subclusters of VMH (Cluster 10).

**Additional file 6: Table S5.** Gene expression profiles of SLC18A3 screened cells.

**Additional file 7: Table S6.** Gene list of three expression modules by pseudotime analysis.

## Data Availability

The datasets generated and/or analyzed during the current study are available in the ArrayExpress repository assigned with the Accession Number E-MTAB-10975 and E-MTAB-10976.
