# Supplementary material for: Transcriptome-scale spatial gene expression in rat arcuate nucleus during puberty
Source: Cell Biosci. 2022 Jan 21;12:8. doi: 10.1186/s13578-022-00745-2 (PMC8781439; doi:10.1186/s13578-022-00745-2)
Supplement: Supplementary file 1 — Additional file 1: Figure S1. H&E staining of ovaries in PND-25, 35 and 45. The solid circular substances indicate corpus luteum. “*” and “**” represent the p-value less than 0.05 and 0.01 adjusted by Student’s t-test. Figure S2. Quality control of spatial transcriptomics sequencing. Figure S3. Top 10 highly-expressed genes in 14 clusters shown by violin plots. Refer to Fig. 2 for the name of 14 clusters. Figure S4. Different cell types shown by feature plots across PND-25, 35 and 45. Figure S5. Expression of Kiss1, Tac3, Pdyn and Slc18a3 shown by feature plots across PND-25, 35 and 45. Figure S6. The top 10 highly-expressed gene of Cluster 9 shown by violin plots in all 14 clusters. Figure S7. The tSNE plot showing the cells are classified into 4 subclusters based on the transcriptomes of overall gene expression relationship among the 1,787 spots of VMH (left). Feature plots showing the distribution of clusters in PND-25, 35 and 45 (right). Different cell clusters are color-coded. Figure S8. The dynamics of spots based on the gene expression profiles along the pseudo-timeline shown by feature plots. Figure S9. The proportion of different cell types in ARC of PND-25, 35 and 45 shown by feature plots. [file 13578_2022_745_MOESM1_ESM.docx]

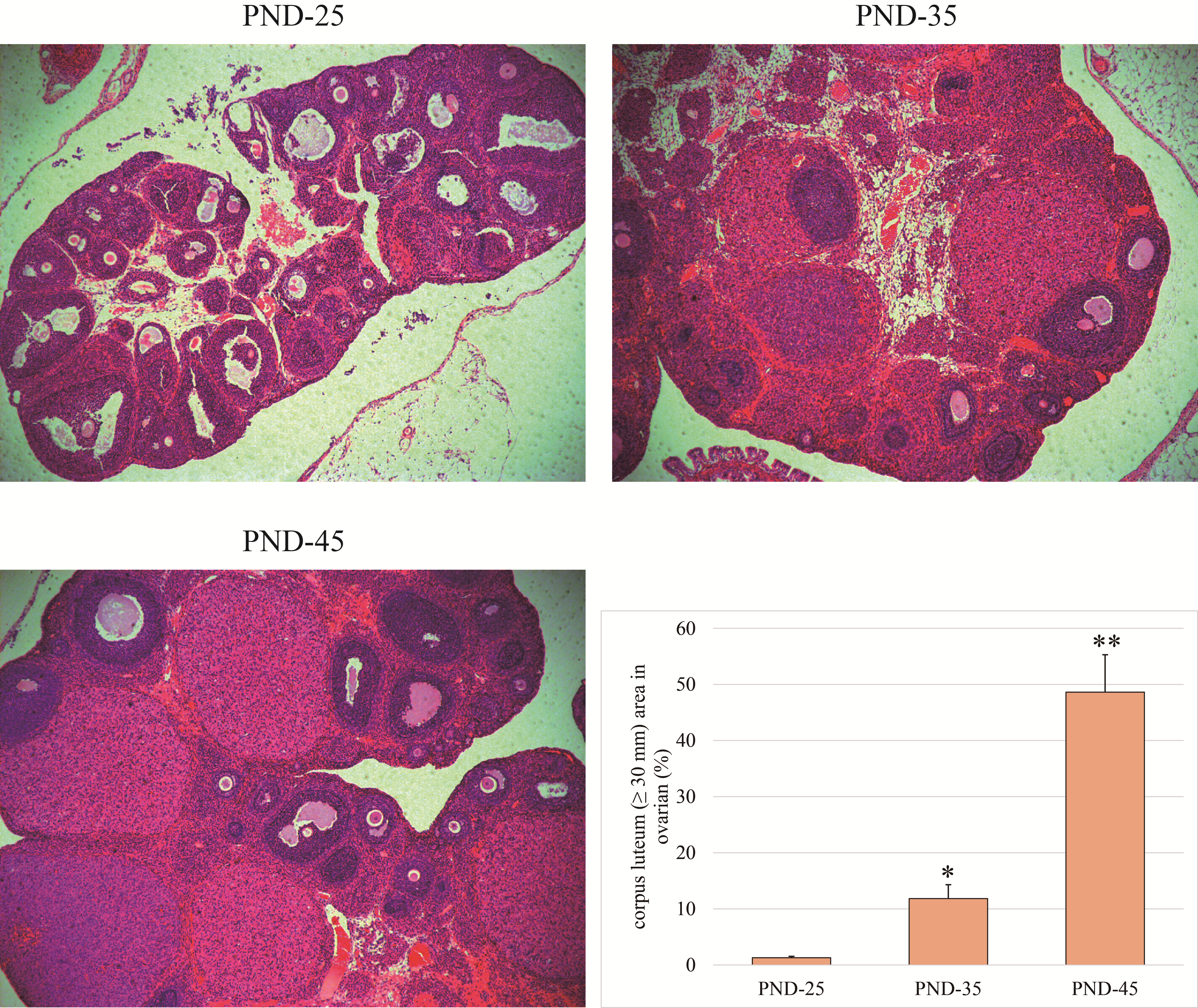


Additional file 1:

Figure S1. H&E staining of ovaries in PND-25, 35 and 45. The solid circular substances indicate corpus luteum. “*” and “**” represent the p-value less than 0.05 and 0.01 adjusted by Student’s t-test.


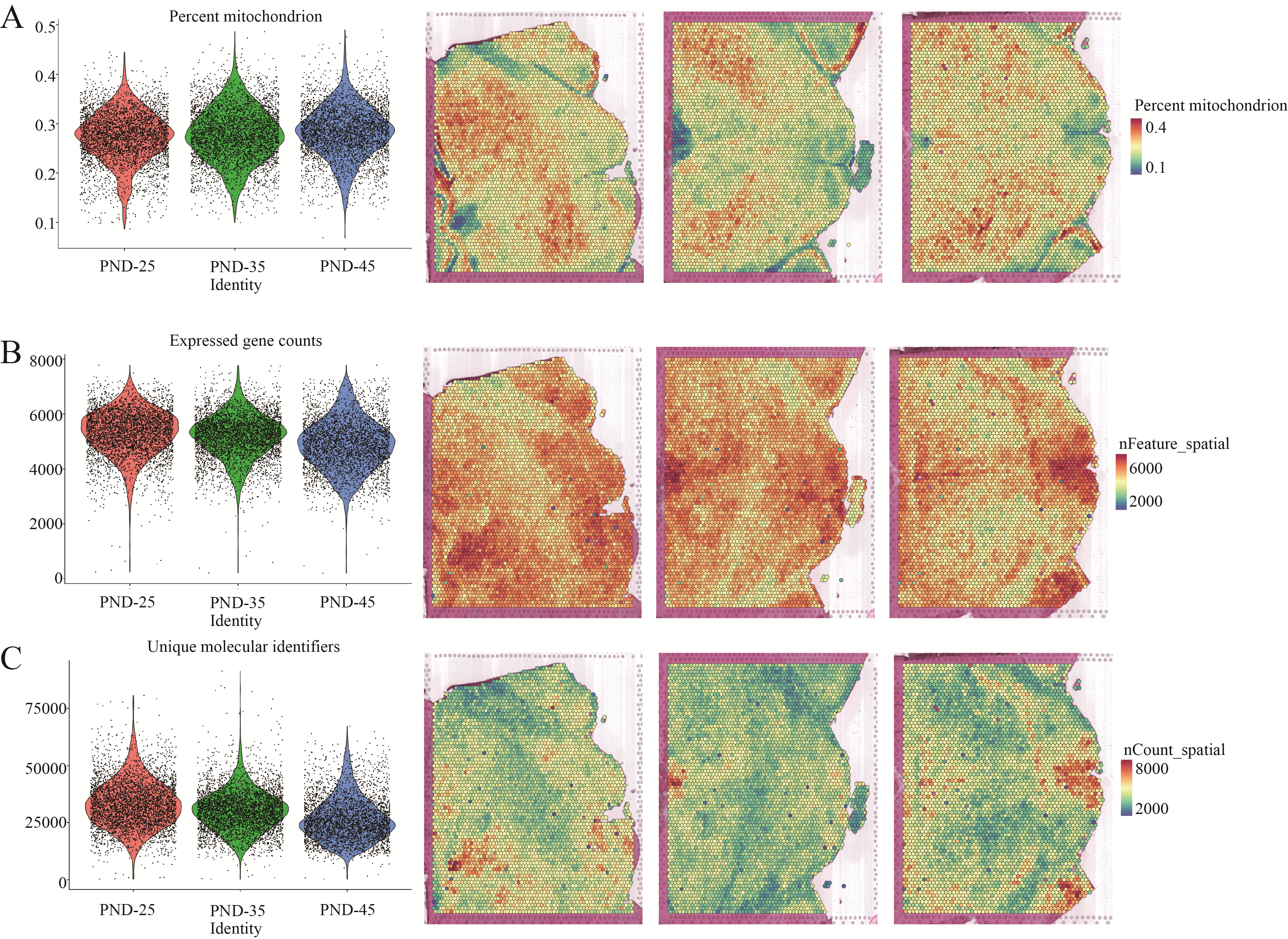


Figure S2. Quality control of spatial transcriptomics sequencing.

1. The proportion of mitochondrial genes in all genes in each spot (left). Feature plots showing the distribution of mitochondrial genes in brain section of PND-25, 35 and 45 (right).
2. The expressed gene counts in each spot (left). Feature plots showing the distribution of expressed gene counts in brain section of PND-25, 35 and 45 (right).
3. The transcript counts in each spot (left). Feature plots showing the distribution of transcript counts in brain section of PND-25, 35 and 45 (right).


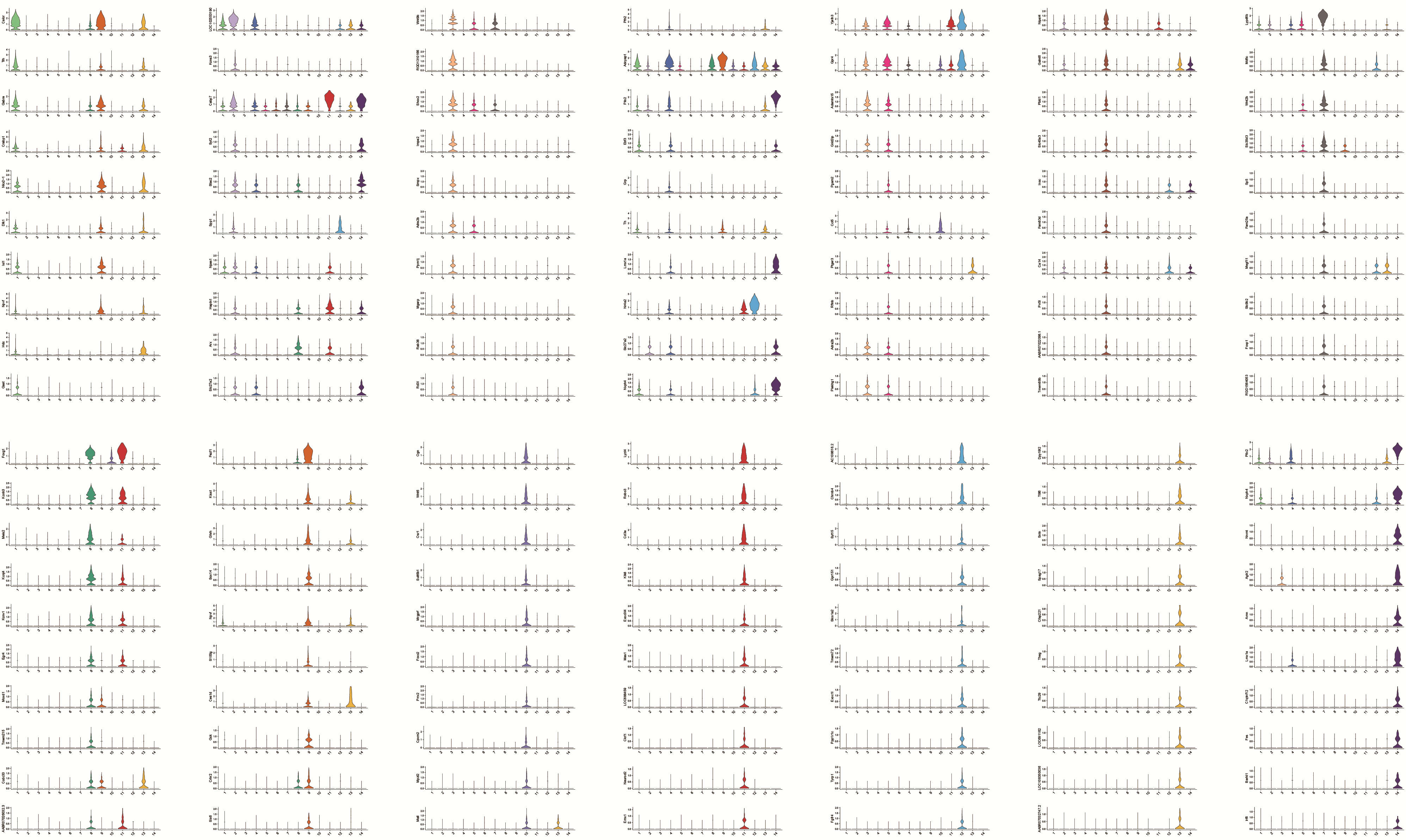


Figure S3. Top 10 highly-expressed genes in 14 clusters shown by violin plots. Refer to Figure 2 for the name of 14 clusters.


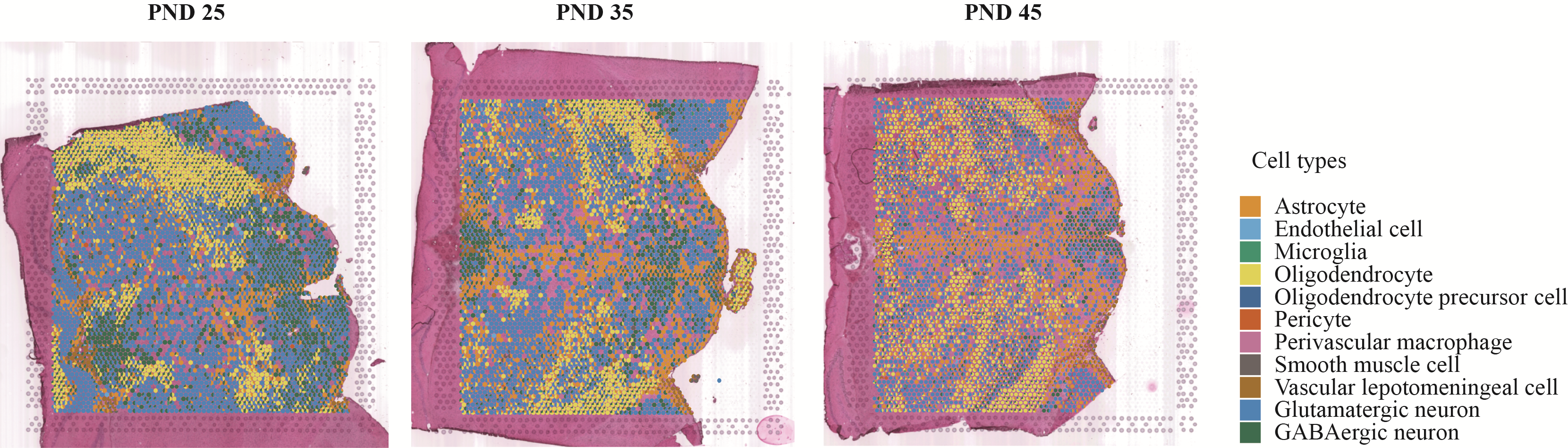


Figure S4. Different cell types shown by feature plots across PND-25, 35 and 45.


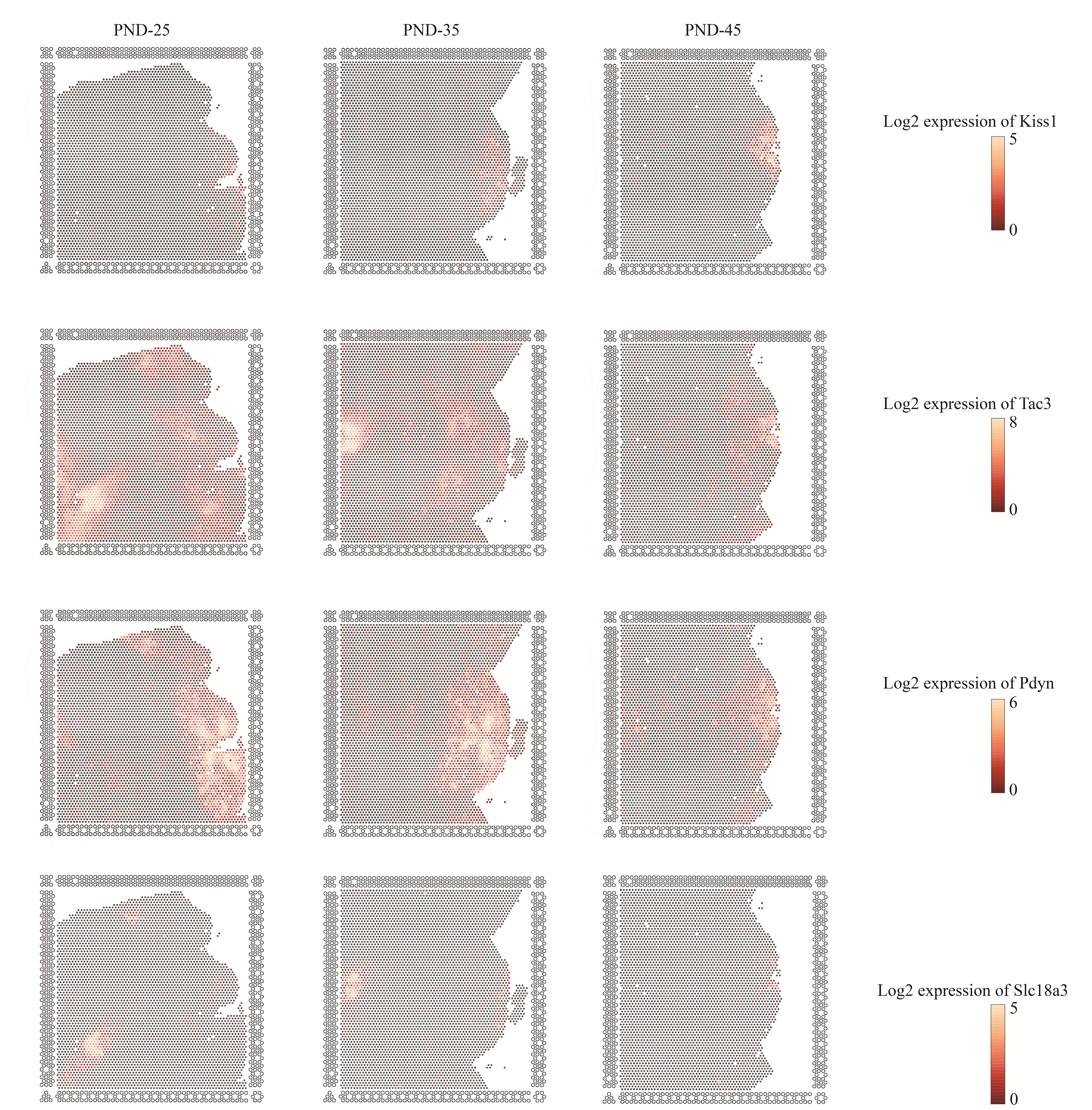


Figure S5. Expression of Kiss1, Tac3, Pdyn and Slc18a3 shown by feature plots across PND-25, 35 and 45.


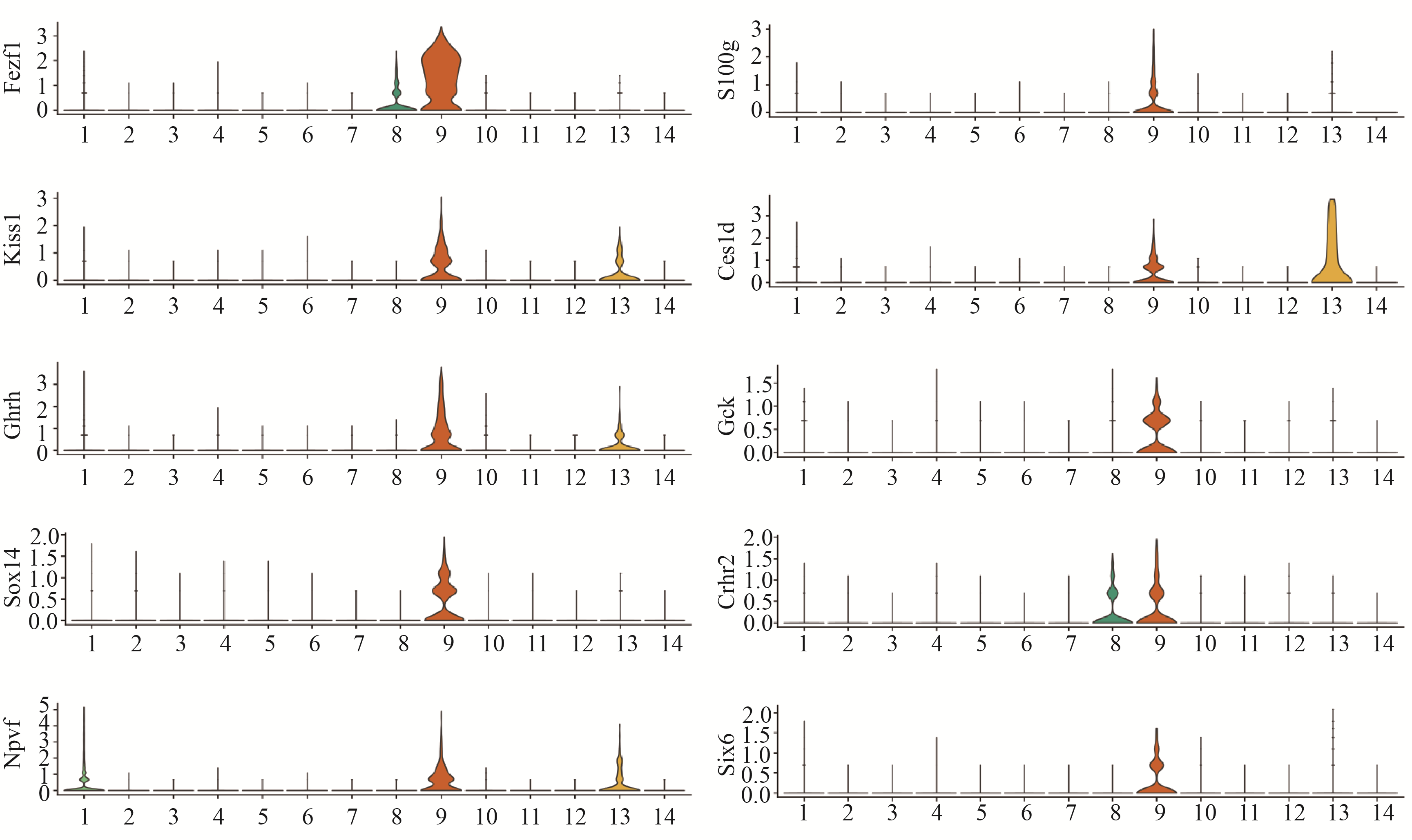


Figure S6. The top 10 highly-expressed gene of Cluster 9 shown by violin plots in all 14 clusters.


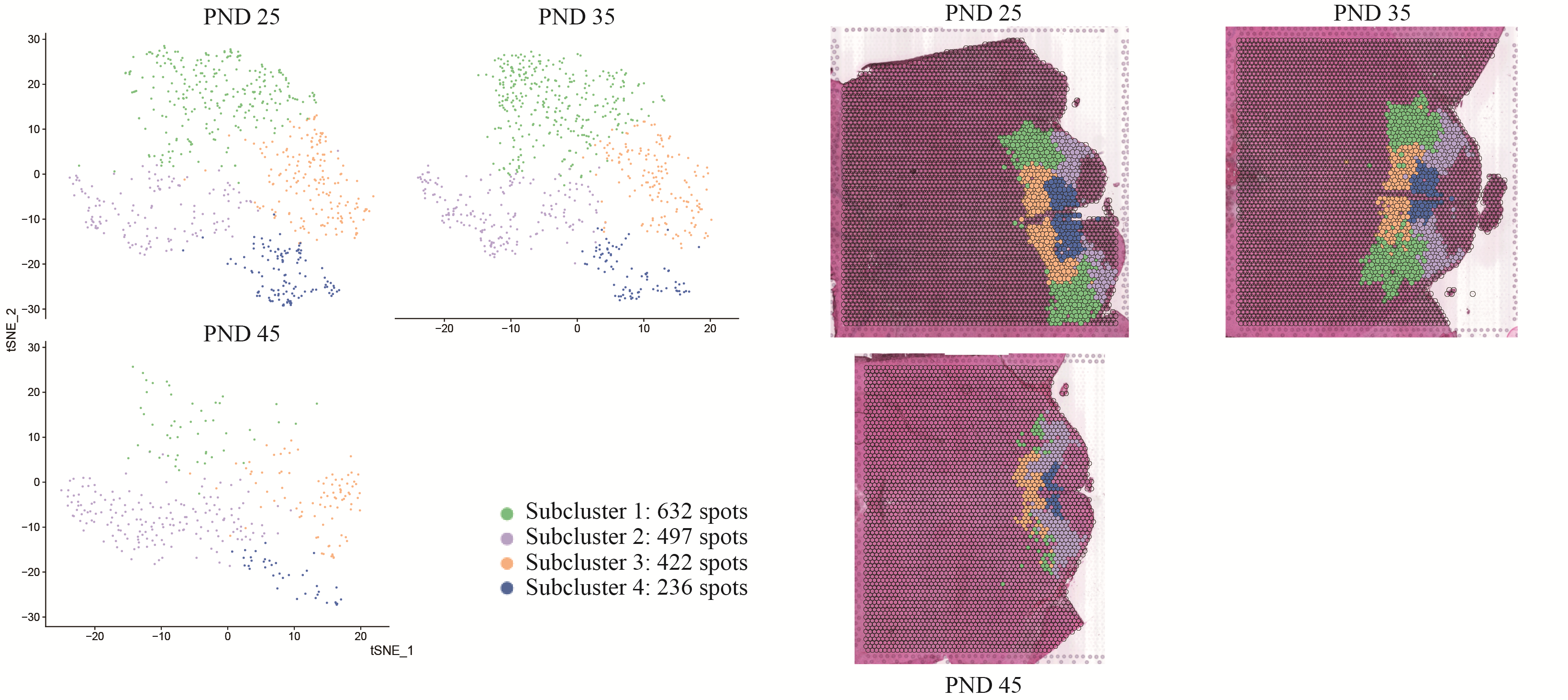


Figure S7. The tSNE plot showing the cells are classified into 4 subclusters based on the transcriptomes of overall gene expression relationship among the 1,787 spots of VMH (left). Feature plots showing the distribution of clusters in PND-25, 35 and 45 (right). Different cell clusters are color-coded.


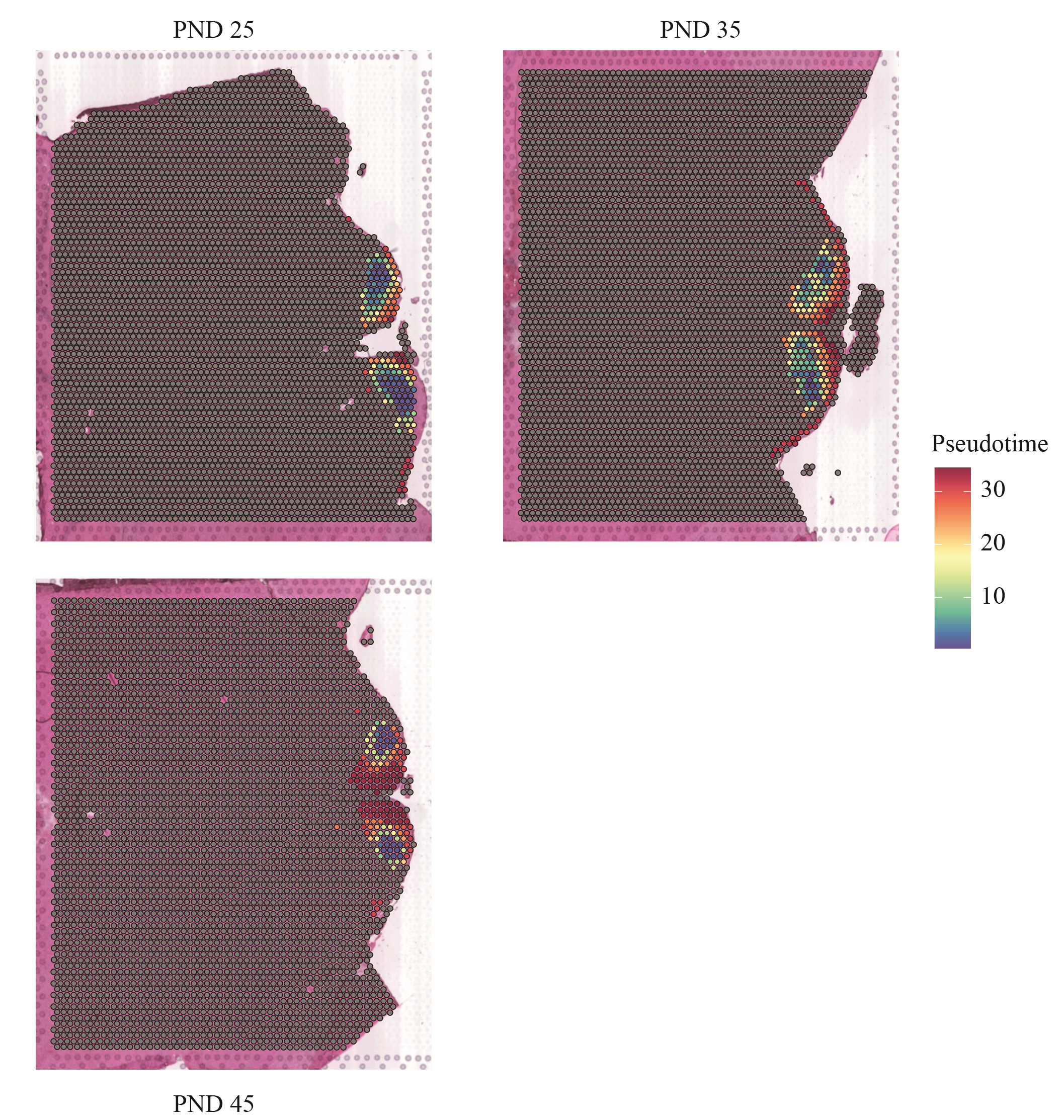


Figure S8. The dynamics of spots based on the gene expression profiles along the pseudo-timeline shown by feature plots.


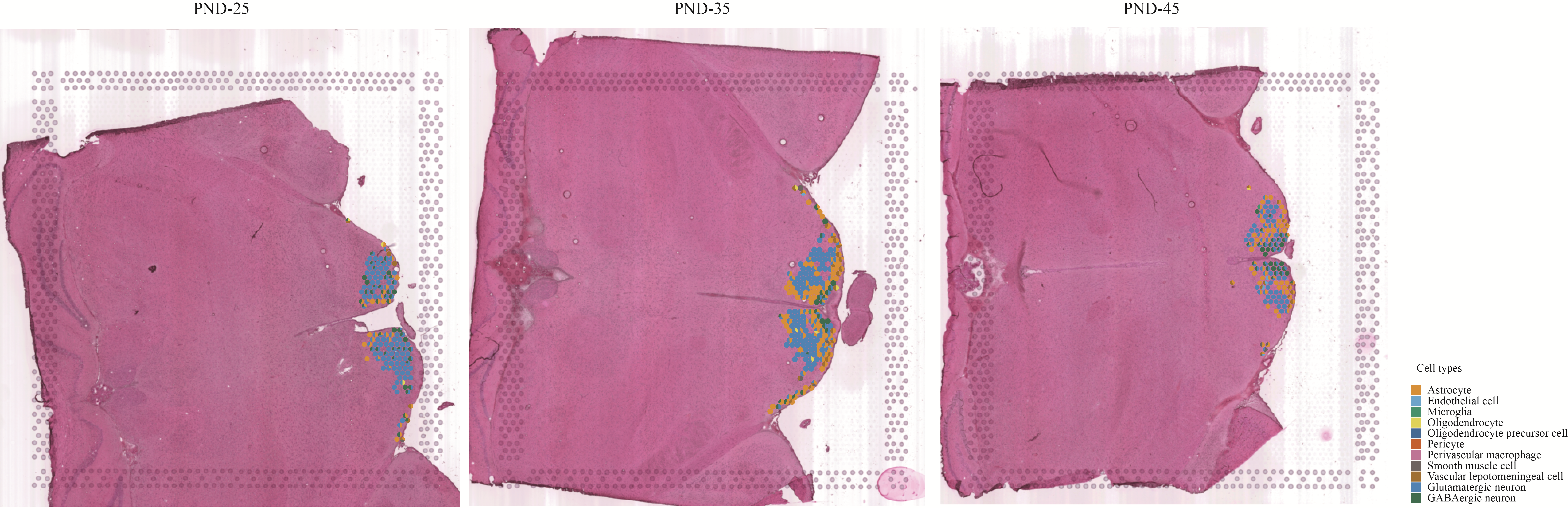


Figure S9. The proportion of different cell types in ARC of PND-25, 35 and 45 shown by feature plots.

**Additional file 2: Table S1.** The basic information of spatial transcriptomics data.

**Additional file 3: Table S2.** Information of DEGs with *p*-value less than 0.01 adjusted by MAST difference test method in all 14 clusters.

**Additional file 4: Table S3.** Information of DEGs with *p*-value less than 0.01 adjusted by MAST difference test method in 4 subclusters of ARC (Cluster 9).

**Additional file 5: Table S4.** Information of DEGs with *p*-value less than 0.01 adjusted by MAST difference test method in 4 subclusters of VMH (Cluster 10).

**Additional file 6: Table S5.** Gene expression profiles of SLC18A3 screened cells.

**Additional file 7: Table S6.** Gene list of three expression modules by pseudotime analysis.
